# Elucidating Poor Decision-Making in a Rat Gambling Task

**DOI:** 10.1371/journal.pone.0082052

**Published:** 2013-12-05

**Authors:** Marion Rivalan, Vincent Valton, Peggy Seriès, Alain R. Marchand, Françoise Dellu-Hagedorn

**Affiliations:** 1 Centre National de la Recherche Scientifique, Aquitaine Institut for Cognitive and Integrative Neuroscience, UMR 5287, Bordeaux, France; 2 Université de Bordeaux, Aquitaine Institut for Cognitive and Integrative Neuroscience, UMR 5287, Bordeaux, France; 3 Institute for Adaptive and Neural Computation, University of Edinburgh, Edinburgh, United Kingdom; Université Lyon, France

## Abstract

Although poor decision-making is a hallmark of psychiatric conditions such as attention deficit/hyperactivity disorder, pathological gambling or substance abuse, a fraction of healthy individuals exhibit similar poor decision-making performances in everyday life and specific laboratory tasks such as the Iowa Gambling Task. These particular individuals may provide information on risk factors or common endophenotypes of these mental disorders. In a rodent version of the Iowa gambling task – the Rat Gambling Task (RGT), we identified a population of poor decision makers, and assessed how these rats scored for several behavioral traits relevant to executive disorders: risk taking, reward seeking, behavioral inflexibility, and several aspects of impulsivity. First, we found that poor decision-making could not be well predicted by single behavioral and cognitive characteristics when considered separately. By contrast, a combination of independent traits in the same individual, namely risk taking, reward seeking, behavioral inflexibility, as well as motor impulsivity, was highly predictive of poor decision-making. Second, using a reinforcement-learning model of the RGT, we confirmed that only the combination of extreme scores on these traits could induce maladaptive decision-making. Third, the model suggested that a combination of these behavioral traits results in an inaccurate representation of rewards and penalties and inefficient learning of the environment. Poor decision-making appears as a consequence of the over-valuation of high-reward-high-risk options in the task. Such a specific psychological profile could greatly impair clinically healthy individuals in decision-making tasks and may predispose to mental disorders with similar symptoms.

## Introduction

Several mental disorders related to poor executive functioning, such as substance abuse, pathological gambling, attention-deficit hyperactivity-disorder or mania, share common deficits and behavioral traits. Impulsiveness, risk taking [1] or inflexible behavior [2], [3], [4], [5], are often present, suggesting that they may jointly contribute to pathological behavior. Poor decision making is a hallmark of these mental disorders as these patients are commonly impaired in the Iowa Gambling Task (IGT). This task measures the capacity to balance risks and gains and to resist immediate gratification in order to receive a larger long-term gain [6]. Interestingly, within a healthy population, a subset of individuals described as impulsive and sensation seekers display poor decision making in this task [7], supporting the notion that a continuum may exist between normality and pathological conditions. Accordingly, neuropsychological characteristics leading to poor decision making in healthy individuals are probably shared by clinical poor decision makers, and could be a potential risk factor for developing related mental disorders [8], [9].

We have developed a single-session Rat Gambling Task (RGT) that reproduces the IGT principles [10], [11], [12]. In this uncertain and conflicting situation, individuals without prior knowledge of the outcomes must gradually learn that the less immediately rewarding options are also less risky and more advantageous in the long term.

Using lesion studies, we have recently shown that good performances in the RGT depend of the functional integrity of several areas of the prefrontal cortex [12]. Like humans, a majority of rats are good decision makers (good DM) and choose the best options, whereas a minority prefers the worst options. These inter-individual differences are stable over time, specific to decision-making processes and reproducible across groups [11]. We previously showed that, like humans, rats that are poor decision makers (poor DM) are risk-prone and more sensitive to reward than good DM [11]. However, although these traits were clearly associated with poor decision making in the RGT, they were not sufficient to dissociate good from poor performers individually, as some good DM were also risk-takers and/or higher reward seekers. Therefore, additional factors, such as inflexibility and impulsivity, could be involved in combination with these traits.

Here, we present an analysis of how inter-individual differences in clinically relevant behavioral traits may contribute to poor and good decision making in the RGT. We show that a combination of several independent behavioral and cognitive characteristics in one individual, namely risk-proneness, motivation for reward, motor impulsivity and behavioral inflexibility, has a cumulative effect and is highly predictive of performance in the RGT. To quantitatively explore the impact of these traits on learning and decision-making, we developed a computational model of the RGT based on the Temporal Difference (TD) learning algorithm [13], [14], [15]. The basic TD framework was extended to take into account risk seeking, reward seeking and cognitive inflexibility and to estimate those behavioral traits in individual rats. The model provides a possible explanation of their impact on learning and decision-making performances in the RGT.

## Materials and Methods

### Ethics Statement

All procedures were conducted in strict accordance with the 2010-63-EU and with approval of the Bordeaux University Animal Care and Use Committee (Permit number: 5012087-A).

### Behavior

**Figure 1:**
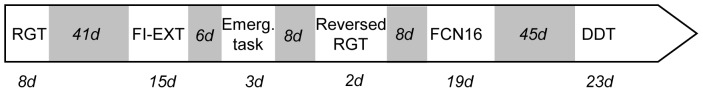
Order and duration of behavioral tasks. The number of days (d) of each behavioral testing phase (below arrow) and inter-test periods (grey zones) are indicated. RGT: Rat Gambling task, FI-EXT: multiple fixed-interval/extinction schedules, Emerg. Task: Light-dark emergence task, FCN16: Fixed consecutive number 16 cue schedule, DDT: Delay discounting task.

**Subjects.** Male Wistar Han rats (*n = *29; Charles River, France) were 12-13 weeks old at the beginning of the experiment. They were housed in groups of four in a temperature (23°C) and humidity-controlled room (60%) on an inverted 12 hr light/dark cycle (lights on at 20:30). Tests were conducted during the dark phase of the cycle. A week before the beginning of the experiments, animals were handled every day. Rats had free access to food and water except during impulsivity and decision-making tests during which they were moderately food deprived (95% free feeding weight). The configuration of the apparatus and the order of testing were chosen to minimize any possible interference between protocols (see Figure 1 for order and duration of tests). The whole behavioral testing phase lasted 6 months (178 days).

**Decision-making.** The RGT requires successive choices among four options in an operant cage [10], [11]. Two of the four options are associated with a higher immediate gain, but are disadvantageous in the long run due to higher unpredictable penalties (time-outs). The experiments were performed in twelve polyvalent conditioning boxes (Imetronic, Pessac, France; 28×30×34 cm). Boxes were equipped with four nose-poke holes, dimly illuminated within the hole with a white LED. These holes were located on a curved wall on one side of the box, equidistant to a food magazine situated on the opposite wall. Each hole was equipped with an infrared detector connected to an external dispenser delivering food pellets (45 mg, formula P, Sandow scientific, USA). Data collection was automated using a control software (Imetronic, Pessac, France) running on a computer outside the testing room. At least thirty minutes before each session, the rats were placed in the light-attenuated and temperature-controlled (23°C) experimental room.

Training: During the training phase, the rats learned to associate two consecutive nose-pokes in one of the four illuminated holes with the delivery of one or two food pellets in the magazine. First, the rats had to associate a single nose-poke in any of the four illuminated holes with the delivery of one food pellet in the magazine. After a nose poke, only the selected hole remained illuminated, but all were inactivated until the rat collected the food reward. This procedure continued daily until rats obtained 100 pellets within a session (30 min cut-off). Then two consecutive nose-pokes in the same hole were required to obtain food, to ensure that the selection of the hole was a voluntary choice. After reaching the same criterion, rats were submitted to two final 15 min training sessions. In the first session, two pellets were delivered after a choice was made (maximum 30 pellets). This session habituated the rats to the quantity of pellets which could be obtained during the test. A second session followed, delivering only one pellet at a time (maximum 15 pellets). The number of reward deliveries was reduced to avoid reduction of sampling and the development of a preference for a hole. The training phase usually lasted 5-7 days and tests were performed the following day.

**Figure 2:**
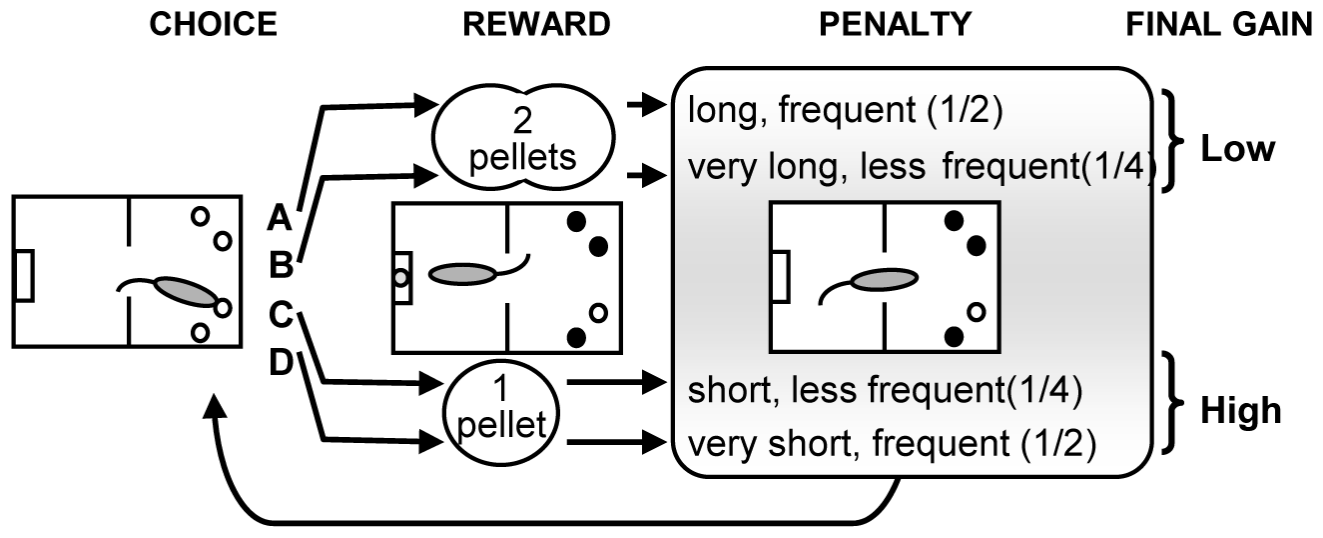
Principle of the Rat Gambling Task. Rats can nose-poke among four different holes (A, B, C and D) in an operant cage, to earn food reward (1-hour test). The selection of one option is immediately rewarded, but can also be followed by a penalty (time-out) of variable duration, according to different probabilities. Two options (C, D) are equally more advantageous than the other two (A, B), which are equally disadvantageous in the long term.

Test: Rats could freely choose between four nose-poke holes (A-D) during a one-hour test session (or max. 250 pellets obtained). Choices C and D *vs* A and B led to the immediate delivery of one *vs* two pellets, but choices A and B could be followed by longer, unpredictable penalties (222 s and 444 s time-outs) compared to choices C and D (12 s and 6 s). Penalties occurred at a low probability (¼) for choices B and C, and at a high probability (½) for choices A and D (Figure 2). During the penalty, all lights were switched off and nose-poke holes were disabled, but the chosen hole remained illuminated to facilitate association between each choice and its consequences. A brief extinction of this light (1 sec) signaled the end of the time-out. The theoretical maximum gain was the same for advantageous choices C and D, and five times higher than for disadvantageous choices A and B.

Good and poor decision makers were differentiated on the basis of the percentage of advantageous choices (>70% and <30% respectively) during the last 20 minutes of test. The remaining rats were undecided with intermediate scores (between 30% and 70% advantageous choices) [10], [11], [12]. The mean latency to collect food pellets after a choice was taken as an indicator of the rats motivation for the food reward [11].

**Behavioral flexibility.** In a second stage, the contingencies for A-B and C-D were spatially reversed to assess behavioral flexibility [11]. To reduce spatial preferences related to the previous experience in the RGT, animals were first given a new training session (100 pellets or 30 min cut-off) during which only one hole at a time, pseudo-randomly, was illuminated and operating at a time, each nose-poke delivering 1 pellet. The test in reversed condition was done the following day, in the same conditions as the RGT, except that options A-B and options C-D were spatially exchanged.

Performances were calculated as the mean percentage of choices for the preferred contingency during the RGT. Behaviors were differentiated on the basis of the time course of choices and flexibility. The observed behaviors were classified into three categories: flexible behavior, with progressive reversion towards the new location of their favorite options (>60% of choices during last 20 min), undecided behavior (choice between 40% and 60%) and inflexible behavior with perseveration of previously learned choices (<40% of choices).

**Impulsive actions: anticipatory hyperactivity and perseveration.** The multiple Fixed-Interval/Extinction schedules of reinforcement (***FI-EXT***) was performed during a single session in operant chambers equipped with one lever. The chambers used for this test were different from the ones used in the RGT [16]. Two periods of fixed-interval schedule of reinforcement (FI) alternated with two periods of extinction (EXT) (FI-EXT-FI-EXT). Impulsive responses corresponded to lever presses during frustrating periods where no reward was available.

The apparatus consisted of eight sound-insulated light-tight outer chambers each containing a two lever conditioning box (Imetronic, Pessac). The boxes (32×32×22 cm) were constructed from white plastic panels with a Plexiglas door. They were equipped with a fan providing a background noise. Each box was permanently illuminated by a diffuse 2 lux light source located in the middle of the ceiling (house light). The floor consisted of 5 mm diameter stainless steel bars spaced 1.5 cm apart. Two stainless steel levers protruded horizontally 1 cm from the wall situated at the left of the door, 16 cm apart and 6 cm above the grid floor. A tray was situated centrally on the opposite wall. Food pellets (45 mg, formula P, Sandow scientific, USA) were delivered in the tray by a food dispenser. A program (Imetronic, Pessac) controlled the chambers and collected the data on a computer situated outside the testing room.

Training and test: During FI, the house light was on and the first lever press after a designated time-interval was reinforced by a food pellet. A light above the lever was on when the pellet was available until the rat visited the tray. During EXT (5 min), the house light was off and no pellet was delivered. During each session, the FI and EXT components operated twice in alternation. Rats were first trained with four sessions with a 30s FI-EXT schedule. Then, rats were trained for four sessions on a 1 min FI-EXT schedule followed by three sessions with a 2 min FI-EXT schedule. A maximum of 7 pellets per FI (14 pellets in total) were delivered during the 1 and 2 min FI conditions. Finally, rats were tested for four sessions on a 1 min FI-EXT schedule to assess adaptability to a change for a shorter FI phase. This latter condition has been chosen for analysis.

Data measure: The mean number of lever presses during each FI and each EXT conditions was recorded. As previously described [16], data from the initial FI after the start of the session, as well as that from the first interval following the first EXT were excluded because the behavior during these intervals might deviate from those during the other intervals. The total mean number of lever press, the number of visits to the empty tray as well as the speed in collecting pellets were also measured for FI and EXT.

**Impulsive actions: premature responses.** The Fixed Consecutive Number of 16 lever press schedule (***FCN16***) measures behavioral inhibition in operant chambers by testing the rat’s ability to carry out a long chain of sequential lever presses before obtaining a reward [17]. The schedule required a fixed minimum number of 16 responses on one lever (FCN lever), signaled by a cue light, before a response on the second lever (Reinforcement lever) resulted in the delivery of one food pellet. Impulsivity was reflected by the proportion of prematurely ended chains of presses on the FCN lever. These chains reset the count and were not rewarded. Chains longer that 16 responses were scored as perseveration.

The operant chambers used for FCN16 testing were similar to the ones used for the FI-EXT schedule, except that they had 2 levers situated on the wall opposite to the food magazine. A cue light above the right lever was also added. The reinforcement lever, much less used than the FCN lever, was the one previously used in the FI-EXT schedule.

Training: On the first day, only the reinforcement lever was available and every press resulted in the delivery of a food pellet in the tray. The rats quickly obtained at least 100 pellets within 40 min (criterion). The following days, both levers were available and the light above the FCN lever was turned on and rats were required to press the FCN lever first and then to press the Reinforcement lever to obtain food (FCN1). The cue light was switched off when the rats had completed the number of consecutive presses required on the FCN lever to obtain food. The cue light signaled the completion of the response requirement to avoid confounds related to time estimation [17].

This cue light was turned on again when rats visited the tray. If the chain was shorter than the number required, the rat had to start a new chain. If the chain was longer, it had no consequence, and the pellet was delivered when the rat pressed the reinforced lever. When 100 pellets were obtained within a session (40 min cut-off), the FCN requirement was progressively increased to 2, 3, 5, and then 8 and 12 using a less strict criterion (45-min cut-off and at least 70 pellets) to avoid overtraining. Rats that failed to reach the criterion in FCN5 after 20 training sessions were excluded from this task. Training under FCN12 lasted a minimum of two consecutive 30-min sessions until rats had reached a stable level of performance.

Test: Rats were tested using the same procedural conditions as in training but with a FCN requirement of 16 lever presses (FCN16) during three consecutive sessions (30 min or 100 pellets cut-off). A rewarded chain of lever presses corresponded to 16 or more lever presses executed on the FCN lever before pressing the reinforced lever.

Data measure: Only data from the third session of FCN16 were analyzed because they revealed the largest inter-individual behavioral differences between good and poor decision makers. Impulsivity in this task is reflected by a low percentage of rewarded chains (<70%). Among rewarded chains, some were just as long as necessary (16 presses) and reflect high response efficiency, whereas some others exceeded the number of presses required and reflect low response efficiency. Thus, response efficiency was estimated by the number of FCN lever presses divided by the total number of food pellet consumed. The number of sessions needed to reach the test phase (learning score) and response rate (total number of each lever responses per min) were also considered. The distribution of the mean number of chain of lever presses according to their length was analyzed.

**Impulsive choice: delay discounting.** The Delay Discounting Task (***DDT***) measures impulsive choice in an operant chamber by assessing the preference for an immediate small reward (one pellet, when pressing one of the levers) over a larger one delivered after a delay (5 pellets, when pressing the other lever). The delay preceding the delivery of the larger reinforcement was progressively increased between sessions.

The operant chambers were the same as those used for the RGT, except that the curved wall was replaced by a straight one equipped with two levers facing the food magazine on the opposite wall. The house light, two cue-lights above the two levers and one cue light in the tray of the food magazine were available and could be turned on and off depending of the procedure.

Training: During training, a press on the right lever (L1) resulted in the immediate delivery of one food pellet whereas a press on the left lever (L5) readily delivered five pellets. Given that the rats were previously trained in the FCN16 schedule that also used two levers (the previous FCN lever being now the L1 lever), a training period was conducted in order to obtain stable performances with no interference from previous requirements. This training period lasted until the rats made more than 70% L5 selections with less than 15% variation in this score on 2 consecutive sessions (in total, 3 sessions were necessary). Whenever an operant lever press was made, a light above this lever was switched on for 1s. Three seconds after food delivery, the magazine light was turned on for 60s, during which time additional presses were without consequence (time-out). The end of this time-out and the beginning of a new trial was signaled by turning off the food magazine light as well as the house light. The duration of the time-out was adjusted such that the duration of each trial was the same whichever lever was chosen.

Test: During the test phase, a press on L1 immediately delivered one food pellet, and was followed by a 60s time-out, whereas a delay was inserted between L5 pressing and the delivery of the five pellets. During this delay, the light above L5 lever remained on until the pellets were delivered, then a time-out (60s minus the length of the delay) immediately followed food delivery. The delay was fixed for a given daily session and increased progressively over the days by 10s from 0 to 40s according to a criterion of stability: scores over two consecutive sessions should not vary by more than 10%. All sessions ended when 100 pellets had been delivered.

Data measures: Percentage of L5 choice, total mean number of lever presses, and presses during the delay and time-out periods were measured. These parameters were calculated for each delay as the mean of the last two stable sessions.

**Risk taking**. The light-dark emergence test allows assessment of spontaneous risk taking behavior in rats [11]. Exiting from a dark, safe compartment to a brightly illuminated one is a risky and stressful situation for a rat. This test was performed in a box (40×40×35 cm) with two small equal compartments that limit exploratory behavior. An aperture (12×31 cm) enabled the rats to pass from one compartment to the other. One was completely enclosed by black opaque plastic sides, with a lid of the same material, while the other was white, had no lid, and was illuminated (560 lux). The rat was placed in the illuminated compartment facing the wall opposite the door. Rat was free to explore the two compartments of the apparatus during a single 10 minute session. Rats were tested in the middle of the dark phase between 10:00a.m and 1:00p.m.

Data measures: From rat first entrance in the dark box, the latency to emerge from this compartment to the illuminated one was recorded (600s cut-off). Risk assessments were evaluated by number of body stretching and by head protruding in the light compartment, with at least the hind limb remaining in the safe compartment. Because these two parameters are correlated with the number of visits of the extremity of the open arms of an elevated plus-maze, which is the more risky area of this task (see [11]), we considered them as a measure of risk-taking. Proportions of visits and time spent in the dark compartment (%) were also measured.

**Analysis of individual differences.** For each test, the proportion of rats with scores above or below the median of the whole population was recorded. These measures were used to compare good and poor DM subgroups and to identify behavioral parameters that could discriminate between the two groups. The scores measured in each of the four individual tasks in which good and poor DM differed were ranked and then summed across the four tasks to produce a global index for each rat.

**Statistical analyses of behavioral data**. Student's t-tests were used to compare subgroup scores in the RGT (mean ± s.e.m.) with indifference level. Comparisons of scores between good and poor decision making groups were made using the non-parametric Mann-Whitney test (*U*). Correlations between scores were evaluated using the non-parametric Spearman correlation test (Statistica, Statsoft 7.1). Comparisons of proportions of individuals were conducted using the non-parametric Fisher exact test (StatXact 9).

### Computational model

**Temporal Difference learning model.** The environment of the RGT was modelled using a Markov decision process. The four possible choices (actions) in the task lead to different rewarded states *s* (i.e. high reward ‘r = 2 food pellets’ for choices A & B or a low reward ‘r = 1 food pellet’ for choices C & D). Each of these states is then followed by a probabilistic transition to the penalty associated with the reward state *s* (penalty transition probabilities are ½, ¼, ¼, ½ for the A, B, C and D states respectively). Penalties correspond to time-outs during which no food can be obtained. In the absence of penalties, rats obtain and consume on average one food pellet in nine seconds (δ_episode_  =  9 s). Therefore, time-outs of duration δ_timeout_(s) can be expressed in terms of a gain loss (in units of food pellets) equivalent to an immediate penalty defined as:
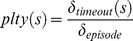
(1)


This results in penalty values of −50, −25, −4/3, −2/3 food pellets for the states A, B, C, and D respectively.

The reward received after taking action *a* in state *s* is described by a state-action pair value Q(s,a), which gradually comes to reflect the 'goodness' of selecting action *a* when in state *s* [22−24]. In this framework, the agent learns the value corresponding to each state-action pair Q(s,a) by updating its expectations of the reward Q(s,a) towards the reward received the last time action *a* was chosen in state *s*. This updating is based on the prediction error between the predicted reward for the state-action pair Q(s,a) and the reward actually received *r*: 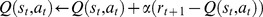
(2)


where α is the learning rate parameter, *r_t+1_* is the reward received after choosing action *a*, Q(s_t_,a_t_) is the current estimate of the value of choosing action *a* in state *s* at time *t* [23]. This learning process causes Q(s,a) to gradually approach the real value of choosing action *a.* No temporal discounting parameter was introduced in this model as individual trials were considered to be independent each of them leading to immediate reward consumption as well as possible penalties.

**Learning model with behavioral traits.** We have extended this basic framework to account for risk seeking, reward seeking, and cognitive inflexibility.

Modeling cognitive inflexibility. The cognitive inflexibility trait is modelled for simplicity by adjusting the learning rate parameter α: α is split into two separate components, an initial learning rate parameter α_ 0_ and an exponential decay with time constant τ_0_, which gradually reduces the learning rate across the session:
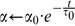
(3)


Parameter α_0_ is comprised between 0 (no learning) and 1. Parameter τ_0_ determines how quickly the agent stops learning and becomes insensitive to the reward prediction error. Each rat is described by particular values of α_ 0_ and τ_0_ and is thus characterised by a unique learning rate profile. Individuals with low α_0_ and/or low τ_0_ describe rats that are inflexible. A further global index of flexibility is given by the integration of α over time. We are aware that recent modelling studies have suggested using a state-splitting mechanism [18], [19] to account for the commonly observed rapid recovery of performances during re-instatement of learned contingencies after extinction. However, our experiments did not address the recovery of the initial RGT conditions after the reversal. Therefore, implementing the state-splitting mechanism would have greatly increased the model complexity (i.e. number of free parameters) without improving the fit to the data.

Modeling reward seeking behavior. The reward seeking trait is introduced as a modulation of the magnitude of the actual rewards r_t_ by a multiplicative weight:
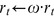
(4)


Values of ω > 1 correspond to the agent representing the reward values as higher than they really are. It was shown experimentally that poor decision makers were able to perform optimally, similar to the good decision makers, in a penalty-only version of the RGT. Therefore, sensitivity to penalty was left constant across animals. In the RGT, rewards are equal to either one or two. Therefore, modelling reward seeking as a multiplicative weight on the true reward provides the simplest way to describe the transformation from objective to subjective reward values [20].

Modeling risk seeking. Following previous work [21], the behavioral trait of risk seeking (or risk aversion) is implemented by adding a positive (or negative) component to the reward that is proportional to the risk level of the action. We define the risk level associated with an action *a* as the standard deviation of penalty values experienced by the agent each time it has taken action *a*:
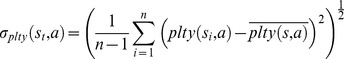
(5)


where *n* denotes the number of times the action *a* was taken from the start of the session and 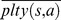 is the average of past penalties: 
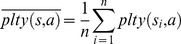
(6)


Therefore, the combination of reward seeking and risk seeking is modelled replacing the reward by: 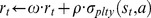
(7)


where *ρ* controls the strength of the risk seeking trait and is unique to each individual rat. A positive value denotes risk-seeking while a negative value corresponds to risk aversion. We choose to model risk in this form, in contrast to some other methods [22], [23], [24], as the present form requires only one parameter and allows learning to reach larger asymptotic values in risky situations.

Final learning model. The resulting model is a TD learning algorithm where risk seeking and reward seeking traits affect the value of rewards, while cognitive inflexibility controls the rate of learning. Putting all the traits together, the learning rule is:
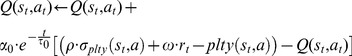
(8)


All actions values are initialised to zero prior to learning.

Decision-making. Actions are selected according to a Softmax process, by assigning a probability of selection to each available action *p(s_t_,a)* depending on the value of all available states:
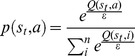
(9)


where *ε* is a temperature parameter which controls the amount of exploration. A high level of exploration is imposed to all subjects during the first 10 min of simulation to ensure that all the options are initially sampled (by analogy with the behavioural procedures).

Parameter estimation & model fitting. The performance of this model during the RGT is fitted to the performance profile of each individual rat using Maximum Likelihood, in order to extract a set of parameters that best describes the rat’s behavior (i.e. a set of four parameters influencing learning *α_ 0_*, *τ*, *ω, ρ* and one parameter influencing the exploration/exploitation trade-off *ε*):
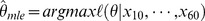
(10)


where 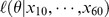
denotes the likelihood of the data under the model, *θ* are the model parameters, and *x_10_* to *x_60_* are the experimental performance levels (percentage of advantageous choices) of the rat over successive 10 min blocks. The likelihood is computed by running the RGT model 50 times for a given set of parameters. Using the performance profiles extracted for each model iteration, we calculate the probability distribution of getting an advantageous choice at every 10 minutes time-bin. The maximum likelihood is the set of parameters that gives the highest probability of resulting in the observed rat performance profile at each of the 10 minutes time-bin.

Model comparison. We used the Likelihood Ratio Test and the Bayesian Information Criterion to test whether simpler models including only 1 or 2 behavioral traits could be as predictive of poor decision making as the full model.

**Data analysis.** The significance of the observed correlation coefficient between the experimental measures and the modeled behavioral traits was tested using Monte Carlo permutations.

Monte Carlo permutation test. This method performs random permutations to mix the paired values (i.e. modelled trait parameter values and the experimental analogue values) and measure the new correlation coefficients for each new permutation. Doing so a large number of times (i.e. 100000 iterations) provides a distribution of correlation coefficients for random permutations of values so as to test the null hypothesis.

Group correlation measure. This correlation measure was used to assess whether the model parameters and experimental measures agreed on the classification of individual rats as having a low or high score for each trait. For each behavioral trait, rats received a score of '-1' (lower than median value for the behavioral trait) or '+1' (higher than median). This was done both for the experimental measures and the estimated parameters. The correlation coefficient between the experimental and theoretical pairs of scores was then computed and the p-value was extracted using the Monte Carlo permutation test.

Individual correlation measure. We also measured whether the estimated model parameters correlated with the experimental measures of reward sensitivity, cognitive inflexibility and risk seeking.

## Results

### Behavior

**Decision-making in the RGT.** The RGT measures, across successive trials, the ability to make the most advantageous choices. In this task, the contingencies associated with a higher immediate gain are disadvantageous in the long run due to higher unpredictable penalties. Decision-making could not be properly measured in six rats because they immediately demonstrated a preference without sampling the different options at the beginning of the test. These rats were discarded from the analysis. Three rats did not display preference for any particular option (undecided subgroup). Because of the small size of this group they were also discarded from our analyses. Among the remaining rats (*n = * 20), behavior during the test was not influenced by prior spatial preference: proportions of individuals with analogous choices during training and testing did not significantly differ from chance (Chi-square test, χ^2^  = .438; *p*  = .33; ns).

**Figure 3:**
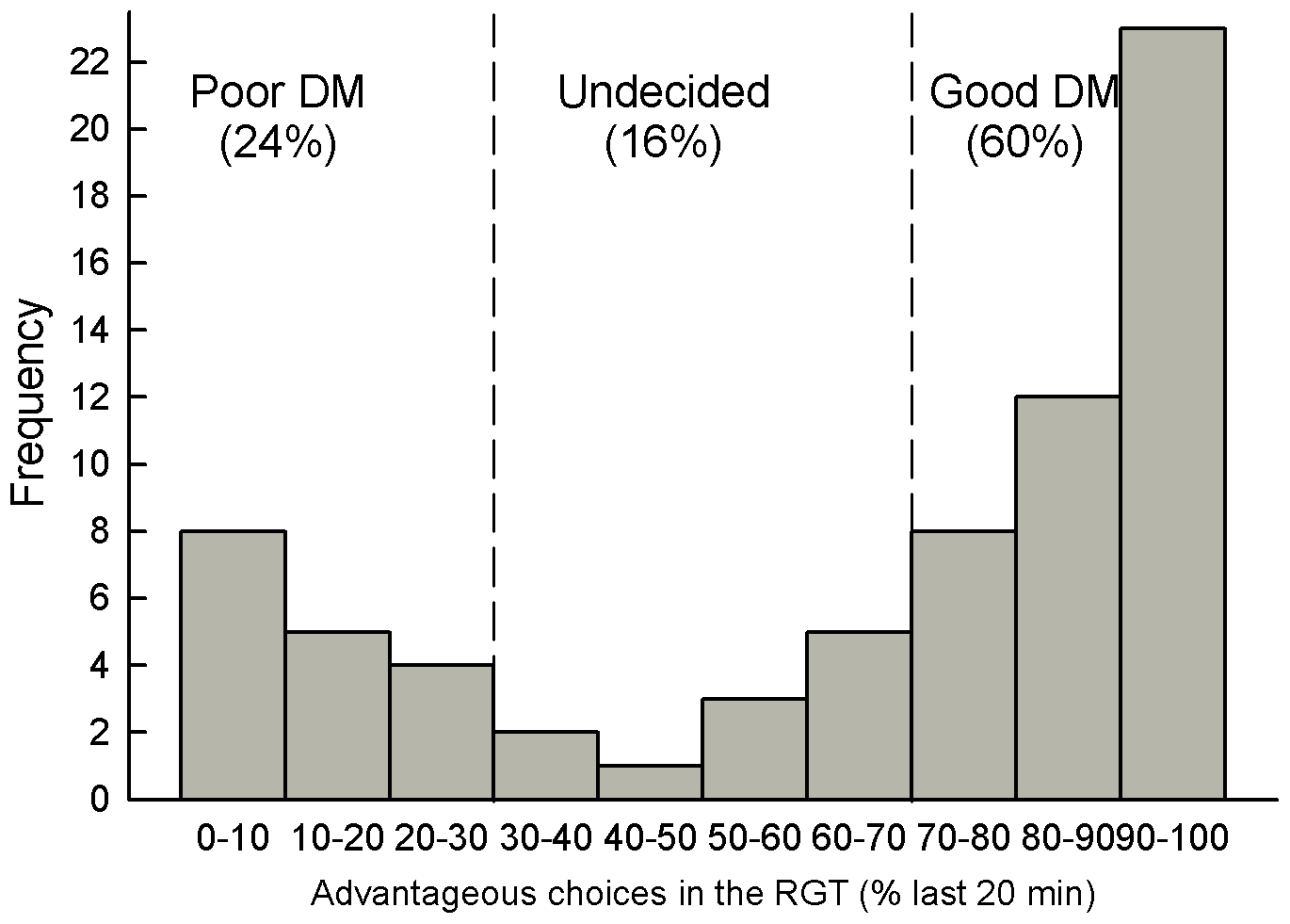
Meta analysis of the RGT data. This analysis is based on 12 distinct experiments (n  =  228) using the same protocol. It reveals a bimodal distribution of RGT scores (% of favourable choices during the last 20 min) with a majority of good decision makers (good DM, with scores above 70%), a minority of poor decision makers (poor DM, with scores below 30%) and the remaining, undecided rats with intermediate scores.

**Figure 4:**
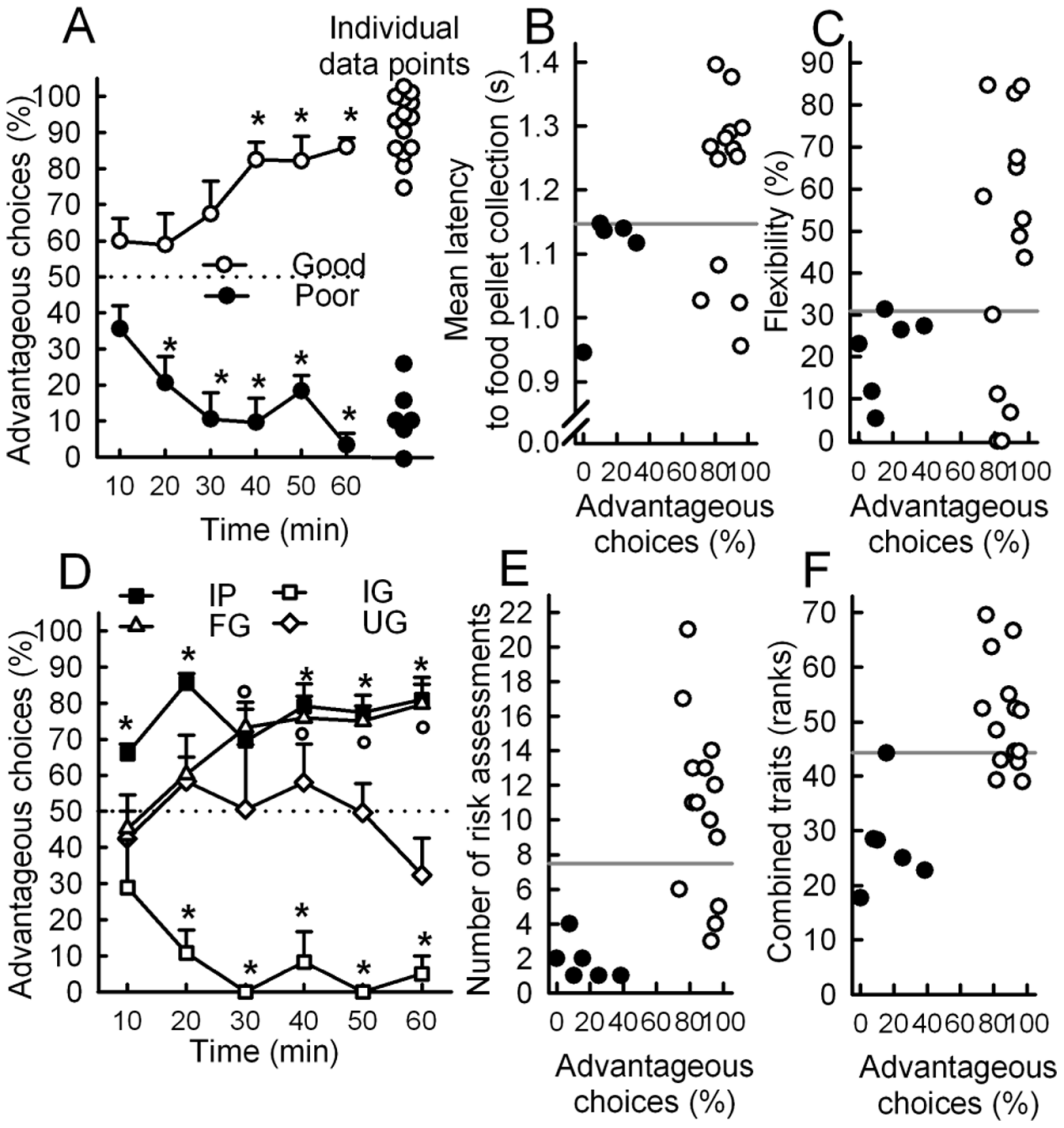
Animal’s performance on the Rat Gambling Task (RGT), RGT-reversed version and the light-dark emergence test. Grey lines represent the median used to compute proportions of high and low scores in good and poor decision makers (DM). (A) Time-course of advantageous choices (%) of good and poor DM on the RGT and individual scores during the last 20 min of good (*n = * 14) and poor (*n = * 6) DM. (B) Relationship between individual RGT scores and the mean latency to collect food pellets (one missing value) during the RGT. (C) Relationship between individual RGT scores and flexibility (final scores in the RGT-reversed version). (D) Time-course of advantageous choices of flexible (FG), undecided (UG) and inflexible (IG) good DM and inflexible (IP) poor DM groups on the RGT-reversed version. Comparison with the indifference level, dotted line, *t*-test: * and ° *p*<.05 at least. (E, F) Relationship between individual RGT scores and (E) the number of risk assessments before the first emergence in the risky compartment, or (F) the individuals’ sum of the score ranks for each behavior.

As observed previously, typical good and poor decision makers can be distinguished within a normal group of rats. Because this task measures a preference between two kinds of options, two subgroups can be easily distinguished, as shown by the bimodal distribution of RGT scores (see meta analysis on Figure 3). Good DM first choose randomly and then gradually orient most of their choices toward the advantageous options (Figure 4A). By contrast, poor DM sample the different options and rapidly orient their choices toward the disadvantageous options (within 10 minutes). During the last 20 minutes, percentages of choices for advantageous options could be divided into two main subgroups: a majority of good DM (*n = *14; 61%, with scores above 70%) and a minority of poor DM (*n = *6; 26%, with scores below 30%) that preferred the disadvantageous options (n.b. scores for the remaining undecided subjects were 38%, 54% and 63%).

**Decision-making and reward seeking.** Poor DM showed a shorter latency to collect their reward than good DM, as previously observed [11] (Figure 4B). All poor DM scores (100%) were below the median *vs* 36% for good DM (Fisher exact test, *p* = .032; group medians, 1.12 and 1.26 s respectively; *U*  = 16, *p*  = .07). However, the global activity of the two groups, reflected by the total number of visits to the nose poke holes, did not statistically differ (median scores: 1025 and 857 for good *vs* poor DM respectively; *U*  = 20, ns).

**Behavioral flexibility.** Reversing contingencies in the RGT measures the rats’ adaptation when advantageous/disadvantageous outcomes are spatially exchanged. Persistence to choose the same location reveals cognitive inflexibility (flexibility <35%), whereas shifting choices reflects detection of the change and behavioral flexibility. All poor DM *vs* only a third (36%) of good DM were inflexible (Fisher exact test, *p = *.014; Figure 4C). Among the remaining good DM, 36% gradually reoriented their choices toward the new location of advantageous options, and 28% distributed their choices between all options (Figure 4D).

**Decision-making and risk seeking.** In the light-dark emergence test, poor DM took more risks than good DM. They emerged more rapidly from the dark compartment than good DM (medians, 35 and 416 sec respectively; *U*  = 13.5, *p*<.02). A majority of poor DM (83%) *vs* 36% of good DM had a score below the median. Poor DM also made much fewer risk assessments than good DM before the first exit (100% *vs* 29% below the median; Fisher exact test, *p  = *.0007; Figure 4E). The median number of risk assessments were 1.5 and 11.2 for poor *vs* good DM respectively (*U*  = 1.5, *p*<.001). Poor DM also tended to make more visits to the bright compartment than good DM (*U*  = 20.5, *p<*.07).

**Decision-making and impulsivity.** Impulsivity is a multifactorial trait encompassing both impulsive actions (inability to delay a response, i.e. premature responses, or to withhold a response, i.e. anticipatory hyperactivity and perseveration) and impulsive choices (inability to wait for a delayed greater benefit) [25].

**Figure 5:**
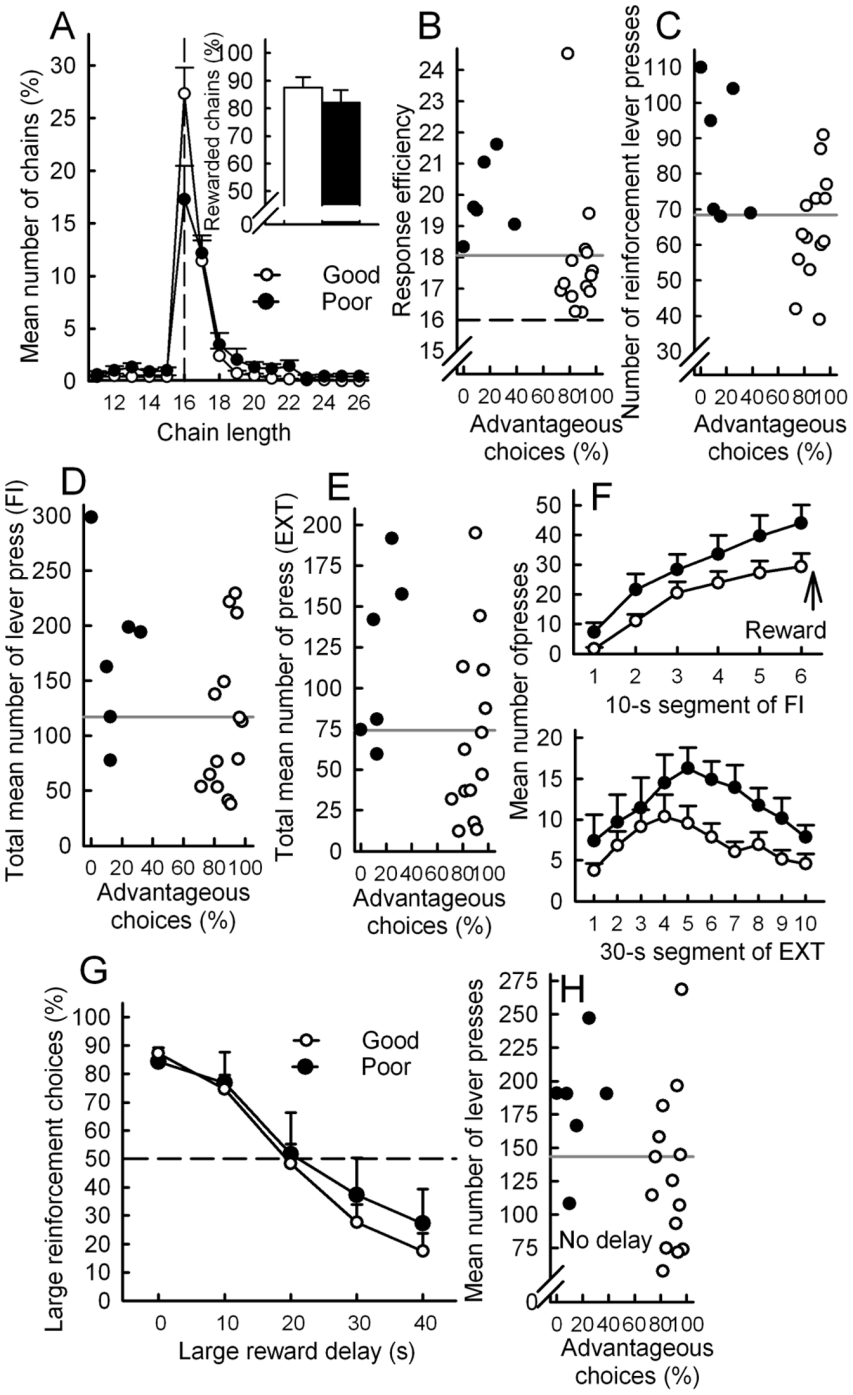
Decision-making and impulsivity. Good and poor decision makers (DM) performances in the (FCN16) Fixed Consecutive Number of 16 lever press schedule (A-C), in the multiple fixed-interval (FI) and extinction (EXT) schedules (D-F) and in the (DDT) delay-discounting task (G-H). Grey lines represent the median used to compute proportions of high and low scores in good and poor DM. (A) Frequency distribution (%) of chain length in the two groups. Optimal chain length (16) is indicated by the vertical dotted line. Inset: Percentage of rewarded chains for good and poor DM (Mean ± SEM). (B,C) Relationship between individual scores in the RGT and (B) response efficiency or (C) the number of reinforcement lever presses. (D,E,F) Relationship between individual scores in the RGT and (D) the mean number of lever presses during the 1-min FI or (E) during the 5-min EXT. (F top panel) Mean number of lever presses of good and poor DM during one 1-min FI component as a function of time. (F lower panel) Mean number of lever presses during the 5-min EXT component as a function time. (G) Percentage of choice for the large, delayed reinforcement as a function of delay in the two groups. (H) Relationship between individual scores in the RGT and the mean number of lever press during DDT training. Dotted line represents chance level.

Impulsive actions: premature responses and compulsive-like behavior. The FCN16 measures response inhibition through the ability to complete a long sequence of lever presses on a first lever (FCN lever) before moving on to another lever (reward lever) that provides a reward [17], [26]. Both groups learned the task at the same rate (learning scores, *U*  = 36, ns). Poor DM did not exhibit any deficit in inhibitory control (i.e. premature switches to the reward lever). The chain length distribution curve of both good and poor DM showed a peak for the optimal chain length (Figure 5A). Both groups predominantly performed rewarded chains (i.e. of length > = 16, Figure 5A-insert). However, poor DM made a higher proportion of long chains of responses (>16), leading to a lower response efficiency (Figure 5B) (*U*  = 8, *p*<.01). The occurrence of very long chains of presses was occasional. For instance, the number of chains longer than 22 presses was 1% of the total number of chains for good DM, and 3% for poor DM. However, all poor DM displayed at least one such very long chain during the test *vs* only 6 out of the 14 good DM. Moreover, whilst the number of presses on the FCN lever did not differ between groups (*U*  = 28, ns), poor DM were more active on the reinforcement lever (*U*  = 18, *p*<.05), making short bursts of presses instead of a single press. These perseverative behaviors, not accompanied by an attempt to collect the reward even when a clear signal announces its availability, are reminiscent of excessive and compulsive behavior. All poor DM had scores on or above the median *vs* 43% for the good DM, which had scores below the median (Fisher exact test *p  = *.018) (Figure 5C).

Impulsive actions: anticipatory hyperactivity and perseveration. The FI-EXT task assesses reward anticipation and sensitivity to context during frustrating periods without reinforcement [16], [27]. Lever press activity is measured either during a delay before a lever press can deliver the reward (FI) or during an extinction phase (EXT) where no reward can be obtained (light house off). During the 1-min FI and 5-min EXT, 83% of poor DM had a motor activity equal to or higher than the median score, *vs* 43% for the good DM (Figure 5D and 5E). Overall, poor DM tended to perform more lever presses than good DM both during FI, (medians, 178 and 98 respectively; *U*  = 23, *p*  = .1) and during EXT (medians, 111 and 55; *U*  = 21, *p  = *.08), suggesting both anticipation and perseveration. Both groups exhibited the typical pattern of activity during each interval of the FI, namely a progressive increase in rate as reinforcement availability approached, with poor DM reaching a score 1.5 times higher than good DM. During EXT, poor performers exhibited both a larger and longer episode of increased activity (Figure 5F). The latency to collect rewards did not significantly differ between groups (*U*  = 31.5), nor did the number of visits to the empty tray (*U*  = 35 and 30, ns). The mean number of lever presses during FI and EXT were positively correlated (*r*  = .69, *p*<.001).

Impulsive choice: delay discounting. The DDT assesses the ability to tolerate a delay when a choice between an immediate small reward and a delayed larger reward is given. It indicates for each individual the subjective value of the large reward as a function of the delay and the delay at which both rewards are perceived to be of equal value. Under the no-delay condition, good and poor DM preferentially chose the larger reward (Figure 5G) and poor DM overall performed more lever presses than good DM (*U*  = 16, *p*<.05, Figure 5H). When the delay increased, both groups shifted to the immediate reward at the same delay, suggesting that they displayed similar reward discounting and tolerance to delay (Figure 5G).

**Correlation between behavioral parameters.** As shown in Table 1, no correlation was observed between reward-seeking, risk seeking and behavioral flexibility. A positive correlation was found between impulsive actions and perseverative responses in different experimental contexts. These parameters (except FI activity) were positively correlated with risk taking, and were independent from inhibitory control capacities (FCN schedule) and impulsive choice (DDT). We decided to model all independent traits (risk, reward and flexibility) excluding motor impulsivity since impulsivity/perseveration measures were correlated with risk seeking (see Table 1).

**Table 1:** Correlations within and between different measures of decision making, flexibility, impulsivity and risk-taking behaviours.

				Reward-seek.	Flexibility	Risk-taking		Inhib. cont.	Motor impulsivity/perseverations			
**Reward-seeking**	RGT	mean latency to collect food	1	-								
**Behavioral flexibility**	RGT-reversal	flexibility index (%)	2	0.09	-							
**Risk-taking**	Emergence	Mean latency to emerge	3	0.11	0.22	-						
	Task	number of risk assessments	4	−0.24	0.11	***0.69	-					
**Inhibitory control**	FCN16	rewarded chains (%)	5	−0.27	0.00	−0.35	0.22	-				
**Motor impulsivity/perseverations**	FCN16	reinforcement lever presses	6	0.14	−0.36	**−**0.51**	*0.45	−0.10	-			
	FI	total lever presses	7	0.22	0.02	−0.28	−0.34	0.09	***0.68	-		
	EXT	total lever presses	8	−0.08	−0.07	**−**0.5**	−**0.52**	0.07	**0.53	***0.69	-	
	DDT	total lever presses (training)	9	0.2	−0.18	**−**0.61**	−**0.53**	0.22	**0.5	0.40	**0.57	-
**Impulsive choices**	DDT	20s-delayed choice (%)	10	−0.23	0.12	−0.26	−0.22	**0.5	−0.10	−0.056	−0.19	0.07

The three behavioral processes included in the model, reward and risk seeking, behavioral flexibility, were unrelated. Impulsive actions and perseverative responses in different experimental contexts were positively correlated. These parameters (except FI activity) were positively correlated with risk taking, and were independent from inhibitory control capacities (FCN schedule) and impulsive choice (DDT). Significant correlations are shown in bold. RGT: rat gambling task; FCN16: fixed consecutive number schedule of reinforcement; FI: fixed- interval; EXT: extinction: DDT: delay discounting task. Pearson’s correlation test; *, *p<*.05; **, *p<*.01; ***, *p*<.001.

**A combination of behavioral traits is highly predictive of poor decision-making**. Poor DM consistently displayed above median scores for each of the following behaviors (Table 2), except one poor DM missing motor impulsivity): motor impulsivity/perseveration, risk proneness, reward seeking and behavioral inflexibility. They obtained a lower global index when these behavioral traits were combined (sum of the ranks) compared to good DM (Figure 4F). By contrast, no good DM ever expressed high scores for more than two of these particular behaviors. Thus, in healthy individuals, the combination of these traits more than any particular one was highly predictive of poor decision making in the RGT. The association of cognitive inflexibility and risk taking behavior or motor impulsivity was never observed in good DM and thus may be a particularly relevant combination of risk factors for impaired decision-making.

**Table 2:** Summary of individual behavioral profiles of poor and good DM.

	Poor decision makers							Good decision makers														
rats	3	8	32	15	28	9	subjects (%)	2	4	24	33	6	31	1	17	19	29	12	30	27	26	subjects (%)
motor impulsivity/persev.	X	X	X	X	X		83	X	X	X			X			X	X			X		43
	X	X	X	X	X			X	X	X			X			X	X					
risk-taking	X	X	X	X		X	83	X		X	X			X		X						14
	X	X	X	X	X	X			X	X	X				X							
reward-seeking	X	X	X	X	X	mis.	100		X		X	X	X		X							36
inflexibility	X	X	X	X	X	X	100	X				X		X				X	X			36
Number of high scores	4	4	4	4	3			2	2	2	2	2	2	1	1	1	1	1	1	0	0	

Motor impulsivity/perseverative responses correspond to high activity scores in both fixed-interval (FI) and extinction (EXT) schedules of reinforcement. Risk taking is indicated by a short latency to emerge and a low number of risk assessment in the dark-light box test, reward seeking by a short latency to collect food in the RGT, and inflexibility by performance in the RGT reversed condition. A cross indicates a high score (with respect to the median) for a given parameter. Last line shows the total number of high scores displayed by each rat. Proportions of subjects demonstrating high score in each group are also given. Mis. : missing value.

### Computational analysis

The TD model was fitted to each rat’s performances in the RGT to estimate the five free parameters describing each rat: two parameters for cognitive inflexibility, one for risk seeking, one for reward seeking and one for the exploration of the environment (see Methods). Partial models with fewer parameters were also tested (see below).

**Figure 6:**
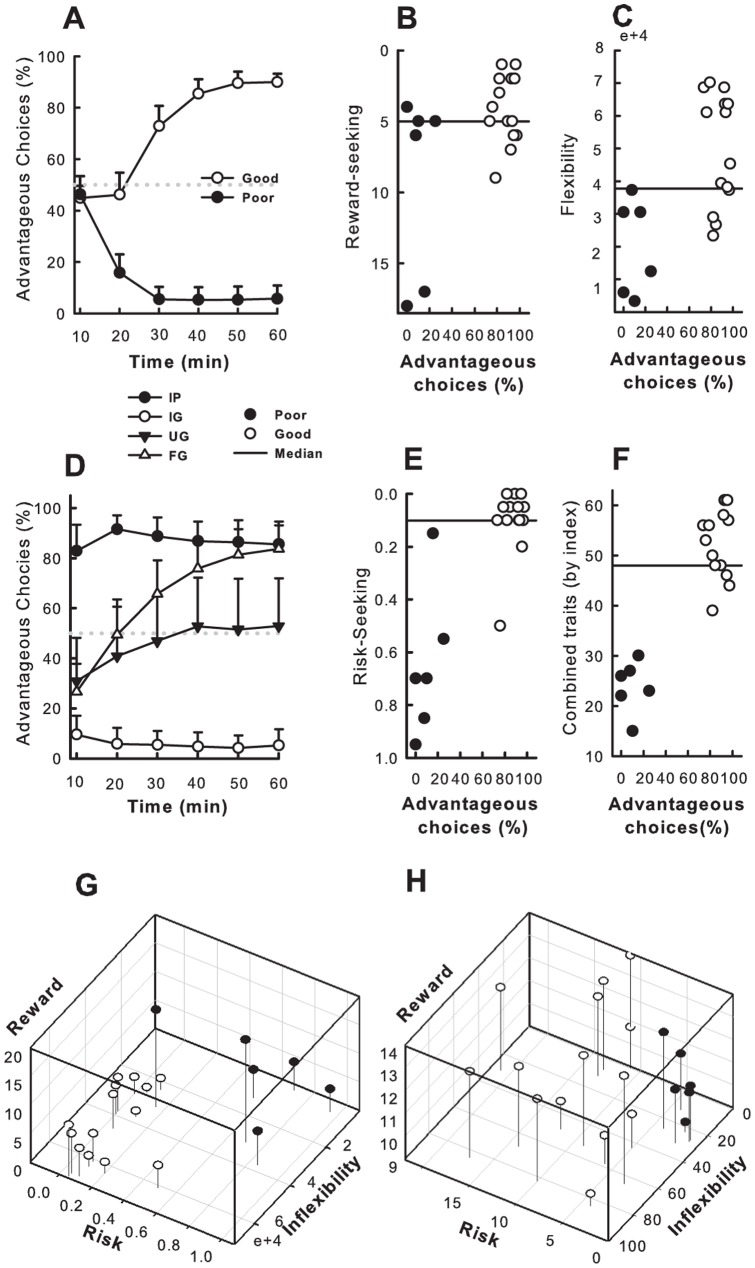
Model’s performance on the RGT, reversal conditions and estimates of individual behavioral levels when fitted to the experimental performance profile of each rat. Grey lines represent the median used to compute proportions of high and low scores in good and poor decision makers (DM). (A) Simulated time-course of advantageous choices (%) of good and poor DM on the RGT. (B) Relationship between simulated individual RGT scores and the estimated reward seeking parameters during the RGT + Reversal. (C) Relationship between simulated individual RGT scores and the estimated flexibility parameters affecting the learning rate. (D) Simulated time-course of advantageous choices of flexible (FG), undecided (UG) and inflexible (IG) good DM and inflexible (IP) poor DM groups on the RGT-reversed version. (E) Relationship between simulated individual RGT scores and the estimated risk seeking parameters. (F) The sum of the simulated score ranks for each modelled behavior. (G) 3-D representation of model parameters for the simulated traits of individual rats. (H) 3-D representation of behavioral measures of the behavioral traits of individual rats.

**Decision-making in the RGT**. The model was able to reproduce the distinct performance profiles observed during the RGT session for poor and good DM (Figure 6A). This suggests that differences in risk-proneness, reward seeking behavior and cognitive inflexibility can collectively account for the variability of performance profiles observed experimentally. Moreover, based on the performance of the rats during the RGT, the model could successfully predict the performance profile of all poor DM and of half of the good DM during reversal conditions (Figure 6D).

**Decision-making and flexibility**. Cognitive inflexibility was implemented as a gradual decrease of the learning rate over the course of the experimental session controlled by two parameters α_0_, the initial learning rate and τ_0_ the decay (see Methods). The initial learning rate parameter α_0_, extracted from a fit of the RGT session alone was positively correlated with the experimental measure of flexibility during reversal (*r* = .3303, group correlation MC permutation test *p*  = .0266). The model predicted an inflexible learning behavior in all modeled poor DM (poor DMm) (Figure 6C), as observed experimentally (Figure 4C). When both the RGT and reversal conditions were used to estimate all model parameters, all flexibility parameters (α_0_, τ_0_ and the area under α) correlated positively with the experimental measure of flexibility (e.g. for α *r*  = −.73, MC permutation test *p  = *.0002, see Figure 6*D*).

**Figure 7:**
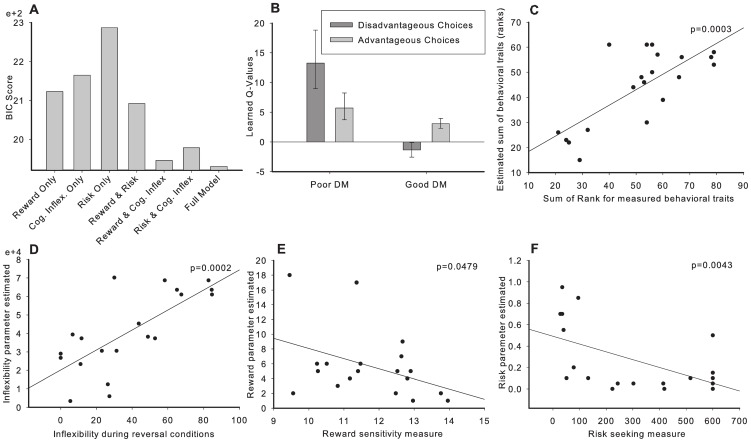
Model comparison and correlations between estimated parameters and behavioral traits. (*A*) Bayesian Information Criterion scores for each model (a low score is better). Models based on two traits fare uniformly better than models based on a single trait. Models with two traits including cognitive inflexibility have better scores than equivalent or simpler models. The model with all three simulated traits provides the best fit to the data even when penalizing for the increased model complexity (number of free parameters). (*B*) Learned Q-Values for advantageous and disadvantageous choices by both Good and Poor DM**.** Bars represent the mean Q-values assigned to the disadvantageous choices (A & B**; dark-grey)** and advantageous choices (C & D**; light-grey**) averaged over all poor or good decision makers at the end of an RGT session. Error bars represent 95% CI around the mean Q-value for all the rats of the population of interest. Poor decision makers vastly over-value disadvantageous choices in comparison to advantageous choices. (*C-F*) Scatter plot illustrating the correlation between: (*C*) The sum of ranks for all the behavioral traits measured experimentally (x-axis) and those estimated by the model (y-axis); (*D*) The measure of cognitive inflexibility (x-axis) and the estimated inflexibility parameter (area under α; y-axis); (*E*) The measured reward sensitivity (x-axis) and the estimated reward sensitivity (y-axis). (*F*) The measured risk seeking (latency to emerge in light compartment; x-axis) and the estimated risk-seeking parameter (y-axis). All estimated parameters correlated significantly with their behavioral counterpart.

**Decision-making and reward seeking.** Reward seeking behavior was modeled by allowing the perceived magnitude of the rewards to be greater than the actual reward. In the model, consistent with experimental data (Figure 4B), all poor DMm except one showed high reward seeking, whereas less than 29% of modeled good DM (good DMm) showed this trait (Figure 6B). The reward seeking parameter estimated from the model correlated significantly with the corresponding behavioural measure of reward sensitivity (*r*  =  −.4014, MC permutation test *p  = *.0479, see Figure 7E).

**Decision-making and risk seeking**. Risk seeking was implemented by adding a risk-related reward contribution [28] to the actual rewards (see Methods). In the model, as in the experiments (Figure 4E), poor DMm were characterized by higher levels of risk sensitivity than good DMm (Figure 6E). The risk parameter extracted from the model significantly correlated with the two behavioural measures of risk seeking (i.e. mean latency for the first visit in the light compartment and risk assessments, *r*  =  −.5370 and −.5555; MC permutation test *p  = *.0043 and *p*  = .0051 respectively, see Figure 7F).

**Combination of behavioral traits**. Finally, when all the different behavioral traits are taken into account (Figure 6F), poor DMm exhibited a combination of high levels for the modeled behavioral traits as observed in behavioural measures. The global index (sum of the ranks of each behavior) of each modeled rat was highly correlated with the global index derived from experimental measures (*r*  = .7420, MC permutation test *p* = .0003, see Figure 7C). Furthermore, similarly to the experimental data (Table 2 and Figure 6H), the model showed that the combination of high cognitive inflexibility, reward and risk seeking is particularly discriminative of poor DMm (Figure 6G), since good DMm almost never expressed more than one of those traits (Table 3).

**Table 3:** Summary of individual modeled behavioral traits of poor and good DM.

	Poor decision makers							Good decision makers														
rats	3	8	32	15	28	9	subjects (%)	2	4	24	33	6	31	1	17	19	29	12	30	27	26	subjects (%)
risk-taking	X	X	X	X	X	X	100			X	X				X	X	X	X		X		50
reward-seeking	X		X	X	X	X	83		X				X	X	X		X		X		X	50
inflexibility	X	X	X	X	X	X	100	X				X				X		X				29
Number of high scores	3	2	3	3	3	3		1	1	1	1	1	1	1	2	2	2	2	1	1	1	

Same representation as in Table 2 for modeled behavioral traits: risk taking, reward seeking and inflexibility parameters.

Influences of combined behavioral traits on Learning. To understand why good and poor DM show different choice preferences, we analysed how well good and poor DMm evaluated advantageous and disadvantageous actions. The Q-values representing the valuation of each choice at the end of the RGT session were extracted for all rats, using the TD-learning model.

Figure 7B illustrates the mean Q-values assigned to the disadvantageous choices (A & B) and advantageous choices (C & D) by poor and good decision makers. Poor DMm vastly over-estimated the value of all states rather than just disadvantageous options. The over-estimation was more important for disadvantageous choices in comparison to the advantageous ones. By contrast, good DMm stopped exploring disadvantageous choices early in the RGT session due to their negative value.

In the model, high scores in risk seeking, reward seeking or inflexibility lead to an altered estimation of the true value of all states. High scores in a combination of traits lead to a shift in the valuation of the state-action pairs, where disadvantageous choices appear to be more valuable than advantageous ones.

**Comparison with simpler models.** Model comparison was also performed in order to address whether simpler models with fewer behavioural traits could have accounted for the experimental data just as well. We tested simpler versions of our model with either only one or two behavioural traits and compared the fit of these models to the experimental data. We used the Likelihood Ratio Test and the Bayesian Information Criterion to assess the fit of the models while penalizing for added complexity. The likelihood ratio test revealed that the full model (including reward sensitivity, risk seeking and cognitive inflexibility) was significantly better (p<0.0001) than any other simpler model, suggesting that all behavioral traits are necessary to describe the experimental data. Similar results were obtained using the Bayesian Information Criterion (See Figure 7A).

## Discussion

Like the IGT in humans, the RGT probably involves a number of cognitive processes, and separating their relative contribution is a challenge. However, our purpose was not to focus on one specific executive function involved in choice, but rather to identify the whole complex phenotype sustaining poor decision making in conflictual and risky situations, as observed in real life. Indeed, a complex interplay between independent behavioral domains is more likely to reflect the complexity of human phenotype and disorders [29], [30], [31].

In the present study, we confirm this hypothesis as we establish a clear link between separate behavioral traits in a normal sample of rats and decision-making in the RGT. Although each trait considered separately has a poor predictive value, both the behavioral and the modeling analyses indicate that poor decision making can be accurately predicted when these traits are considered in combination.

While integrating multiple cognitive abilities, the RGT offers the advantage to assess the time-course of the decision making process within a single session. It is particularly suitable for identifying inter-individual differences in decision making, and notably for identifying poor decision-makers because choices are made readily and lead to two opposed decisions: either a preference for advantageous options or a preference for the disadvantageous ones [11]. As shown by the meta-analysis of several experiments in the RGT, these behaviors are reproducible. Importantly, poor decision-making does not result from a slower learning. We have previously shown that repeating the RGT on three consecutive days does not change the rats’ preferences (data not shown). Additionally, acquiring information about the value of the options separately before the test does not change the behavior of poor and good decision-makers, nor does it change their relative numbers [11].

We show that poor decision making is expressed by individuals presenting excessive scores for a combination of behavioral and cognitive traits: risk taking, higher reward seeking behavior, motor impulsivity and behavioral inflexibility, expressed simultaneously. This contrasts with good DM which present a wider range of scores and only express up to two of these characteristics (Table 2). The various traits that we examined were largely independent from one another. A noteworthy exception was the relationship between motor impulsivity/ perseveration and risk taking (see Table 1).

Poor DM are characterized by risk and reward seeking, which have been found to be associated with trait dominance in rats and humans, and could be necessary for the development and maintenance of social structure [32], [33]. Interestingly, risk and reward seeking, in combination with impulsivity, are hallmarks of poor decision making related mental disorders such as ADHD [34], personality disorders, substance abuse [28], [35], pathological gambling [36] or mania [37]. Poor DM are also characterized by behavioral inflexibility as well as perseverative and compulsive-like behaviors. Their inflexibility was particularly noticeable in the RGT reversal procedure, which requires redirecting choices on the basis of new response-reward contingencies [38], but also in the FCN schedule with perseverative responses. Indeed, perseverative responses in the FCN have similarly been observed following amphetamine administration (0.8 mg/kg), in a similar procedure [39]. These effects of the psychomotor stimulant are likely to reflect compulsivity, especially at this dose, given that only low doses of amphetamine (0.25 mg/kg) are known to reduce impulsivity in this task [17], [26], whereas higher doses (0.5 mg/kg or above) increase impulsive responses. Perseverative behavior, typically observed after acute administration of psychostimulants [39], inflexible and compulsive behavior can be seen in drug addiction [40], [41], pathological gambling [2] and in obsessive-compulsive disorder (OCD) [1]. Inappropriate compulsive behaviors [25] may result from attributing excessive incentive value to reward associated stimuli [42], [43]. This could explain bursts of activity on the reinforcer level in the FCN schedule, as well as hyperactivity in the FI-EXT schedule. Compulsive behavior could also result from a quicker switch from initial voluntary goal-directed behavior to an habitual, automatic process with loss of control, as observed in drug addiction and OCD [44], [45]. Interestingly, poor decision-makers do not have more impulsive tendencies compared to good DM in terms of intolerance to delayed gratification and of inhibitory control. Still, we cannot exclude that more demanding tasks (e.g. the stop-task [46]) could reveal differences in inhibition between both phenotypes. Moreover, the higher sensitivity of poor DM may have influenced the performance in this task. However, a recent meta-analysis also concluded that inhibition and decision-making in the IGT are dissociated [47].

Previous studies have shown that individual behavioral traits can be related to maladaptive behavior in animal models of mental disorders (i.e. novelty-seeking in depression [48]; impulsivity, novelty preference in drug self-administration [49], [50], [51]). However, the cumulative effect of several symptoms in one individual, as systematically observed in mental disorders [1], has rarely been considered in an animal model [29]. Here, we show that a complex phenotype is highly predictive of poor decision-making, since it only describes poor performers. Each of the traits identified participates to this phenotype that leads to the inability to adapt to the situation because of a distorted representation of the balance between reward and risk, and an inflexible/compulsive behavior precluding readjustment of behavior. This complex phenotype reflects well the relevance of the concept of “domain-interplay” to explore the basis of maladaptive behavior [29], [30]. Although we cannot conclude that the different observed phenotypes observed represent innate or acquired differences, it is noteworthy that dominant rats are natural risk takers and display increased motivation for food reward [32], [33], two characteristics of poor decision makers in the RGT. This social parameter could be well related to performance in the RGT, a hypothesis that remains to be elucidated.

Recent experiments based on lesion studies have shown that good performances in the RGT depend of the functional integrity of the prefrontal cortex, notably the prelimbic, cingulate and orbitofrontal cortices [12]. Moreover, the brain networks differentially activated during adaptive and maladaptive decision-making reveal striking differences that can be related to the behavioral and cognitive traits identified (manuscript submitted) [52].

Building on the expanding literature indicating that behavioral traits such as risk seeking affect learning and the prediction error signal [20], [53], we used a reinforcement learning model of the RGT to investigate the relationship between the traits and the decision making performances. First, we used the model to address whether the behavioral traits could collectively account for the variety of performances observed in the decision-making task (i.e. Can excessive behavioral traits lead to poor and/or undecided decision-making?). Secondly, we used the model to explore the interaction between the behavioral traits on learning and decision-making (i.e. How and why do excessive traits lead to poor decision-making?). The computational model, based on a TD-learning algorithm [54], [55], [56] was modified to include the behavioral traits of risk seeking, reward sensitivity and behavioral inflexibility.

The model reveals how risk seeking, reward sensitivity and behavioral inflexibility jointly contribute to the learning and the decision-making process. The model of the RGT fits the experimental data very closely (Figure S1), and demonstrates that behavioral traits of high risk seeking, high reward seeking and cognitive inflexibility can be derived from the performance of individuals in the RGT. Importantly, all the parameters used to model the behavioral traits successfully correlated with the experimental measures for each trait, validating the assumptions made during the implementation. This suggests that the mathematical formalization of all the behavioral traits and their independent influence on learning in the RGT were valid. Interestingly, we found that individual traits were insufficient to lead to poor performances at the task (Table 3). Rather, poor decision-making required specific combinations of at least two of the behavioral traits, namely inflexible learning and risk seeking or inflexible learning and reward seeking. This suggests that single excessive behavioral traits may be compensated for in good decision makers. Yet, such potential compensatory processes may fail when a combination of traits are involved.

Importantly, the computational study is based on the assumption that a failure in decision-making occurs through an altered internal representation of the values in the environment (Figure 7B), as is customary in computational modeling of psychopathology [57], [58]. We investigated the difference in valuation of the different choices by poor and good decision makers. Surprisingly, we found that poor DMm vastly over-estimate the value of all choices, but especially those corresponding to disadvantageous options. According to their inflated valuation of disadvantageous choices, poor DM appear to behave optimally according to their inaccurate value-map of the environment, rather than sub-optimally according to the objective outcome of the task. Our findings are in line with recently suggested mechanisms of psychopathology such as addiction [53].

Our model accounts for the role of behavioral traits in learning and decision-making, using a basic TD-learning framework using minimal assumptions. Other formalisms such as win-stay loose-shift, Bayesian models or more elaborate TD models could also be explored [18], [19], [20], [22], [23], [24]. However, the present model offers a straightforward way to implement the traits of interest and allows a quantitative assessment of the impact of individual differences on the overall decision-making performances. In particular, we show that simple models incorporating fewer discriminative traits have less predictive value than the full model. More biologically targeted versions of this model could be developed [59], [60], [61] and investigated with regard to the cortical- subcortical interplay specific to good and poor DM [52].

In conclusion, poor decision making in the RGT is predicted by a complex phenotype of cumulated behavioral and cognitive characteristics including risk seeking, reward seeking and inflexibility, combined with motor impulsivity and perseverative/compulsive-like behaviors. This approach, based on the identification of high scores for these behavioral traits expressed spontaneously and in a comparable way as to those observed in the clinic, demonstrates that rat behavior can reliably model dimensions found in humans [8], [62]. This work emphasizes the need to use “integrative” animal models to mimic the complexity of the clinically relevant phenotype [30]. Our findings are also in line with the recent proposal by Robbins et al. [31] to undertake a more objective description of psychiatric disorders through predisposing traits and neurocognitive endophenotypes, thereby explaining the high level of comorbidities between mental disorders. By integrating multiple behavioral measures, combined with computational modeling, our work provides a promising framework for revealing the neuropsychological determinants of poor decision-making as a potential risk factor for developing related mental disorders [8], [9] and for exploring its neurobiological substrates.

## Supporting Information

Figure S1 **Models’ best fit to individual rat performances**. Each graph shows the performance of the rat (dashed-line) in terms of % of advantageous choices (y-axis) over time (x-axis). The model mean performance (continuous line) and standard deviation (grey area) is represented on the same graph for each rat.
(TIF)
