# Supplementary figures and images for: Elucidating Poor Decision-Making in a Rat Gambling Task
Source: PLoS One. 2013 Dec 5;8(12):e82052. doi: 10.1371/journal.pone.0082052 (PMC3855331; doi:10.1371/journal.pone.0082052)

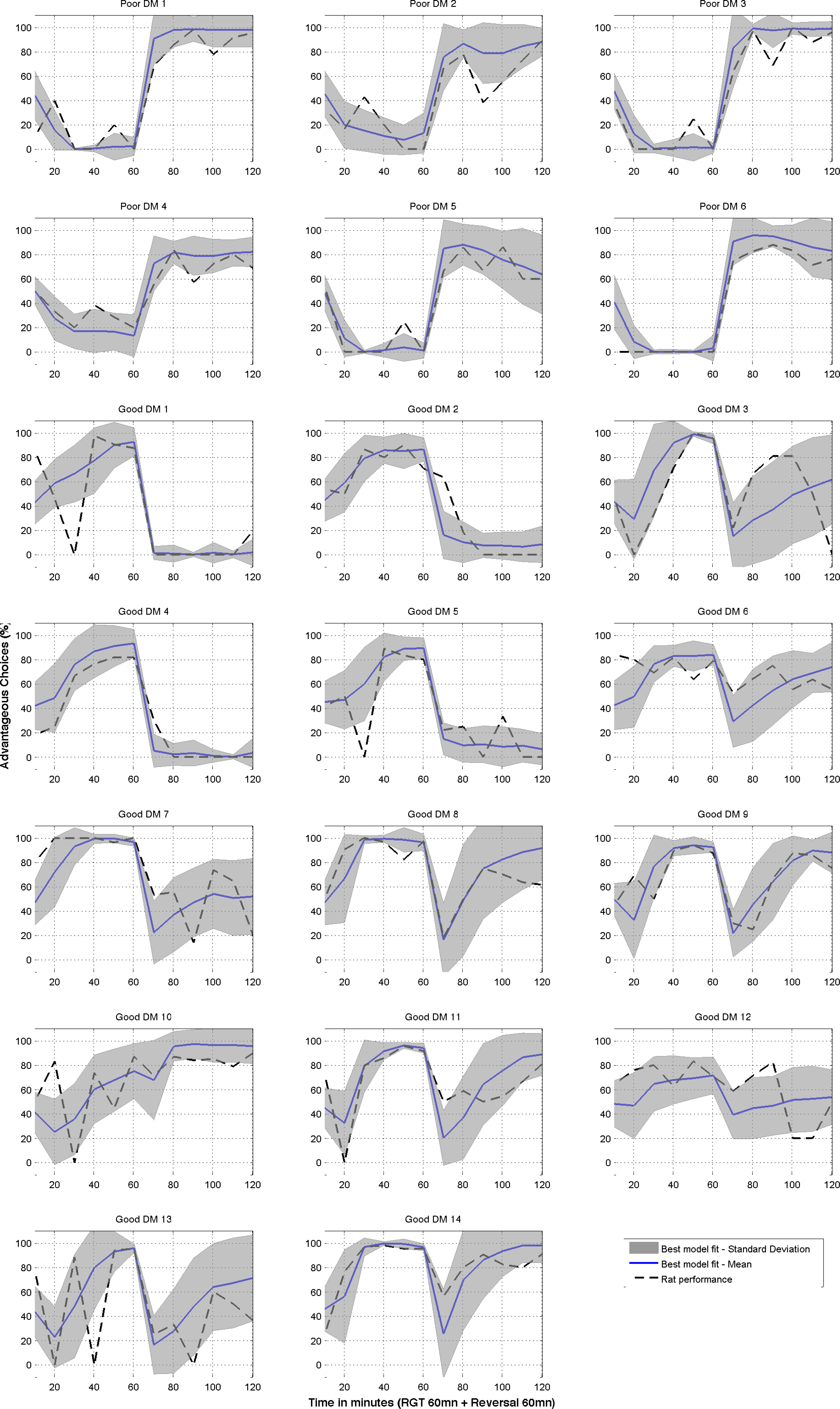

Supplement: Figure S1 — Models’ best fit to individual rat performances. Each graph shows the performance of the rat (dashed-line) in terms of % of advantageous choices (y-axis) over time (x-axis). The model mean performance (continuous line) and standard deviation (grey area) is represented on the same graph for each rat. (TIF) [file pone.0082052.s001.tif]
